# Everolimus-eluting bioresorbable scaffolds and metallic stents in diabetic patients: a patient-level pooled analysis of the prospective ABSORB DM Benelux Study, TWENTE and DUTCH PEERS

**DOI:** 10.1186/s12933-020-01116-2

**Published:** 2020-10-02

**Authors:** T. M. Hommels, R. S. Hermanides, B. Berta, E. Fabris, G. De Luca, E. H. Ploumen, C. von Birgelen, E. Kedhi

**Affiliations:** 1 Isala Hospital, Zwolle, The Netherlands; 2 Cardiovascular Department, University of Trieste, Trieste, Italy; 3 AOU Maggiore della Carità, Eastern Piedmont University, Novara, Italy; 4 Medisch Spectrum Twente, Thoraxcentrum & University of Twente, Thoraxcentrum, The Netherlands; 5 Department of Cardiology, Hôpital Erasme Université Libre de Bruxelles, Brussels, Belgium

**Keywords:** Bioresorbable scaffolds, Drug-eluting stents, Diabetes mellitus, Coronary artery disease, Percutaneous coronary intervention, Device thrombosis

## Abstract

**Background:**

Several studies compared everolimus-eluting bioresorbable scaffolds (EE-BRS) with everolimus-eluting stents (EES), but only few assessed these devices in patients with diabetes mellitus.

**Aim:**

To evaluate the safety and efficacy outcomes of all-comer patients with diabetes mellitus up to 2 years after treatment with EE-BRS or EES.

**Methods:**

We performed a post hoc pooled analysis of patient-level data in diabetic patients who were treated with EE-BRS or EES in 3 prospective clinical trials: The ABSORB DM Benelux Study (NTR5447), TWENTE (NTR1256/NCT01066650) and DUTCH PEERS (NTR2413/NCT01331707). Primary endpoint of the analysis was target lesion failure (TLF): a composite of cardiac death, target vessel myocardial infarction or clinically driven target lesion revascularization. Secondary endpoints included major adverse cardiac events (MACE): a composite of all-cause death, any myocardial infarction or clinically driven target vessel revascularization, as well as definite or probable device thrombosis (ST).

**Results:**

A total of 499 diabetic patients were assessed, of whom 150 received EE-BRS and 249 received EES. Total available follow-up was 222.6 patient years (PY) in the EE-BRS and 464.9 PY in the EES group. The adverse events rates were similar in both treatment groups for TLF (7.2 vs. 5.2 events per 100 PY, p = 0.39; adjusted hazard ratio (HR) = 1.48 (95% confidence interval (CI): 0.77–2.87), p = 0.24), MACE (9.1 vs. 8.3 per 100 PY, p = 0.83; adjusted HR = 1.23 (95% CI: 0.70–2.17), p = 0.47), and ST (0.9 vs. 0.6 per 100 PY, p > 0.99).

**Conclusion:**

In this patient-level pooled analysis of patients with diabetes mellitus from 3 clinical trials, EE-BRS showed clinical outcomes that were quite similar to EES.

## Background

Diabetes mellitus is a well-established predictor of adverse clinical and angiographic events following percutaneous coronary intervention (PCI) with metallic drug-eluting stents (DES) for obstructive coronary artery disease (CAD) [1–6]. Various disease-related factors contribute to a pro-inflammatory and pro-thrombotic state that reduce the prognosis of patients with diabetes mellitus, such as endothelial dysfunction, changes in plaque composition, platelet activation and coagulation disturbances [7]. Following PCI with stent implantation, the aforementioned mechanisms may be even aggravated by the permanent presence of the metallic vascular prosthesis that mechanically distorts the arterial geometry, delays vascular healing and constrains vascular response in the treated coronary segment [8–10].

The everolimus-eluting bioresorbable scaffolds (EE-BRS) were developed to overcome some shortcomings of the metallic DES [11, 12]. Because of its resorbable nature and its transient presence after implantation, it was hypothesized that EE-BRS may be associated with a more favorable restoration of the treated coronary vessel and a more favorable long-term clinical outcome. In addition, the use of EE-BRS may allow for repeating PCI in the same target lesion without loss of lumen size due to the lifelong presence of multiple layers of metallic stents in the coronary vessel. Furthermore, it is likely to facilitate coronary artery bypass grafting (CABG) that may be required at a later point in time. Consequently, patients with diabetes mellitus, who are known to have an increased risk of repeated PCI and finally surgical treatment with CABG, may show a particular benefit from PCI with the utilization of EE-BRS.

Nevertheless, data from studies that compare EE-BRS and DES in an all-diabetic patient population are scarce. For that reason, we pooled patient-level data from 3 prospective clinical trials to compare the clinical outcome after PCI with EE-BRS and everolimus-eluting stents (EES) in all-comers with diabetes mellitus.

## Methods

### Study population

For the purpose of this analysis we pooled data of all patients with diabetes mellitus, who were treated with EE-BRS in de novo coronary lesions in the prospective ABSORB DM Benelux Study or who underwent PCI with EES in de novo coronary lesions in the TWENTE and DUTCH PEERS trials. The design and outcomes of the individual studies have been reported previously [13–16]. The ABSORB DM Benelux Study is a prospective, international observational study in all-comer patients with diabetes mellitus who underwent PCI with EE-BRS. The TWENTE study is a Dutch randomized trial that examined the performance of second-generation EES to zotarolimus-eluting stents in all-comers. DUTCH PEERS is a Dutch multicenter randomized trial that assessed the performance of new-generation EES to zotarolimus-eluting stents in an all-comer population. All available follow-up data up to 2 years after PCI was used.

### Percutaneous coronary intervention procedure

Implanted EE-BRS devices were the bioresorbable polymer drug-eluting scaffold ABSORB BVS system and the ABSORB GT1 system (Abbott Vascular, Santa Clara, CA, USA). Both devices are composed of poly-L-lactic acid with a strut thickness of 150 µm, covered by a polymer coating of poly-DL-lactic-acid that elutes everolimus. EE-BRS are expected to be completely resorbed within 3 years [12]. The available scaffold diameters ranged from 2.50 to 3.50 mm with lengths of 8–28 mm. The durable fluoropolymer-coated Xience V EES (Abbott Vascular, Santa Clara, CA, USA) is a cobalt-chromium EES with a strut thickness of 81 µm; it was available in diameters from 2.25 to 4.00 mm with lengths of 8 to 28 mm. The strut thickness, polymer coating, eluted drug and available stent diameters of the platinum-chromium-based Promus Element EES (Boston Scientific, Natick, MA, USA) were the same as for Xience V EES; the available stent length ranged from of 8 to 38 mm.

Angiographic success was defined as a < 50% residual stenosis of the target lesion after successful device implantation, as visually assessed. Procedural success was defined as angiographic success without occurrence of any adverse cardiac event during index hospitalization. In general, dual antiplatelet therapy (DAPT) was prescribed for 12 months after PCI, reflecting contemporary international guideline recommendations.

### Study endpoints

The primary device-oriented endpoint of target lesion failure (TLF) is a composite of cardiac death, target vessel myocardial infarction (TV-MI) or clinically driven target lesion revascularization (TLR). Secondary endpoints include the patient-oriented endpoint of major adverse cardiac events (MACE), which is defined as a composite of all-cause death, any myocardial infarction (MI) or clinically driven target vessel revascularization (TVR), as well as definite or probable device (i.e., scaffold or stent) thrombosis (ST). All endpoints were defined according to clinical data standards of the American College of Cardiology/American Heart Association and the Academic Research Consortium [17–20].

### Statistical analysis

Categorical variables are presented as frequencies and percentages. A 2-sided Pearson’s Chi square test was performed to determine significant differences between the EE-BRS and EES groups. The Fisher’s exact test was used if appropriate due to small sample sizes with low frequency variables. Continuous variables with normal distribution are presented as mean and standard deviation, and a 2-sided independent student t-test was used to compare groups. An equal variance was upheld unless a significant Levene’s test had been calculated; in that case a t-test with unequal variances was conducted. The clinical outcomes of the composite endpoints TLF and MACE were obtained by means of Kaplan–Meier survival methods with time-to-event analysis in addition to a log-rank test at 2-year follow-up. To adjust for variable time to follow-up between trials, all endpoints and the composites of TLF and MACE are presented in event rates per 100 patient years (PY) with the Poisson distribution given with 95% confidence interval (CI). In addition, a multivariate Cox regression model with adjustment for age, insulin-treated diabetes mellitus, number of treated vessels, total treated vessel length and treatment group was built for both TLF and MACE, reported as hazard ratio (HR) and 95% CI. P-values < 0.05 were considered statistically significant. Statistical analyses were performed using SPSS version 25 (IBM Corp., Armonk, NY, USA).

## Results

Patient-level data were available from 499 diabetic patients; 150 patients (188 target lesions) were treated with EE-BRS and 249 (336 target lesions) with EES. Baseline clinical characteristics of both patient groups are shown in Table 1. Patients treated with EE-BRS were younger and had less frequently a family history of cardiovascular disease. Other baseline characteristics, including previous ischemic heart disease, the prescription of insulin and the clinical presentation at the time of the index PCI did mostly not differ between the groups. The incidence of chronic kidney failure (serum creatinine levels of ≥ 130 µmol per liter), as determinant risk factor for cardiovascular disease, was limited and also did not differ between both study populations.

**Table 1:** Clinical characteristics of the patients at baseline

Baseline clinical characteristic	EE-BRS (n = 150)	EES (n = 249)	p-value
Age (years)–mean ± SD	64.3 ± 10.1	67.1 ± 10.2	<0.01
Sex (male)–no.(%)	108 (72.0)	159 (63.9)	0.09
Body-mass index (kg/m^2^)–mean ± SD; *no.*	29.5 ± 5.1, *148*^a^	29.3 ± 4.8, *221*^a^	0.63
Risk factors–no.(%)			
Insulin-treated diabetes mellitus	52 (34.7)	163 (34.5)	0.98
Arterial hypertension	104 (69.3)	176 (70.7)	0.78
Hypercholesterolemia	100 (66.7)	157 (63.1)	0.47
Family history of cardiovascular disease	59 (39.3)	131 (52.6)	0.01
Current smoker	35 (23.3)	50 (20.1)	0.44
Medical history–no.(%)			
Previous acute coronary syndrome	28 (18.7)	59 (23.7)	0.24
Previous PCI	37 (24.7)	54 (21.7)	0.49
Previous CABG	8 (5.3)	24 (9.6)	0.13
Chronic renal failure^b^	6 (4.0)	12 (4.8)	0.70
Clinical presentation–no.(%)			
Acute coronary syndrome	73 (48.7)	120 (48.2)	0.93
Myocardial infarction	47 (31.3)	74 (29.7)	0.73
Unstable angina pectoris	26 (17.3)	46 (18.5)	0.77
Non-acute coronary syndrome	77 (51.3)	129 (51.8)	0.93

Shown are the clinical characteristics at baseline for both patient groups

*EE-BRS* everolimus-eluting bioresorbable scaffolds, *EES* everolimus-eluting stents, *PCI* percutaneous coronary intervention, *CABG* coronary artery bypass grafting

^a^Plus–minus values are mean ± standard deviation (SD) and the number in italic^a^ represent the known total of which the variable was calculated

^b^Renal insufficiency was defined as serum creatinine level of ≥ 130 µmol per liter. A p-value < 0.05 was considered as statistically significant

The angiographic characteristics are presented in Table 2. There was no significant difference in the distribution of the target vessels, except for left main stem treatment which was only performed in a few EES-treated patients. In the EE-BRS group, a few patients with CABG treatment were included. Although the number of target lesions per patient was similar in both groups, the EES group had a greater number of treated vessels, which resulted in a longer total device length. In addition, the EES group underwent more often bifurcation treatment. Nevertheless, both treatment groups showed similar angiographic and procedural results.

**Table 2:** Angiographic characteristics of the patients at baseline

Baseline angiographic characteristic	EE-BRS	EES	P-value
Patient-level analysis			
Number of patients	150	249	
Number of treated vessels–mean ± SD	1.1 ± 0.3	1.2 ± 0.4	<0.01
Number of treated target lesions–mean ± SD	1.3 ± 0.5	1.3 ± 0.6	0.22
Total number of devices–mean ± SD	1.4 ± 0.7	1.8 ± 1.1	<0.01
Lesion-level analysis			
Number of lesions	188	336	
Coronary artery lesion distribution–no. (%)			
Right coronary artery	57 (30.3)	108 (32.1)	0.67
Left anterior descending artery	89 (47.6)	144 (42.9)	0.42
Circumflex artery	40 (21.3)	82 (24.4)	0.30
Left main	0 (0)	10 (3.0)	0.02
Arterial or venous graft	2 (1.1)	0	0.13
Coronary artery lesion characteristics			
Bifurcation or trifurcation–no. (%)	27 (14.4)	77 (22.9)	0.02
Device-level analysis			
Number of devices	214	454	
Diameter device–mean ± SD^b^	3.0 ± 0.4	2.9 ± 0.5	<0.01
Highest device inflation–mean ± SD; no.^c^	14.3 ± 2.6, *211*^*a*^	15.4 ± 4.3, *446*^*a*^	<0.01
Total length of devices per lesion–mean ± SD^b^	23.7 ± 11.7	27.0 ± 15.4	<0.01
Procedure-level analysis			
Number of PCI procedures	188	336	
Results–no. (%)			
Post-procedural TIMI grade 3	186 (100), *186*^*a*^	334 (99.4)	0.54
Angiographic success	185 (100)*, 185*^*a*^	334 (99.4)	0.54
Procedural success	184 (97.9)	327 (97.3)	0.70
Peri-implantation procedures			
Predilatation–no. (%)	177 (94.1)	227 (67.6)	<0.01
Predilatation balloon size–mean ± SD; *no*.^b^	2.8 ± 0.8, 176*	2.4 ± 0.5	<0.01
Predilatation pressure–mean ± SD; *no*.^c^	14.8 ± 4.0, *174*^*a*^	14.5 ± 4.5	0.51
Postdilatation–no. (%)	142 (75.5)	273 (81.3)	0.12
Postdilatation balloon size–mean ± SD^b^	3.2 ± 0.5	3.1 ± 0.5	0.34
Postdilatation pressure–mean ± SD^c^	17.3 ± 4.3	22.5 ± 5.5	<0.01

Shown are the angiographic characteristics for both patient groups

^a^Plus–minus values are mean ± standard deviation (SD) and the numbers in italic^a^ represent the known total of which the variable was calculated

^b^Length of lesions, devices and balloons were measured in millimeter (mm) as was the diameter of the devices

^c^Dilatation and inflation pressures were measured in atmosphere (atm)

A p-value < 0.05 was considered as statistically significant

*EE-BRS* everolimus-eluting bioresorbable scaffolds, *EES* everolimus-eluting stents, *PCI* percutaneous coronary intervention, *TIMI* Thrombolysis in Myocardial Infarction with grade 3 referenced as completely restored flow

**Fig. 1:**
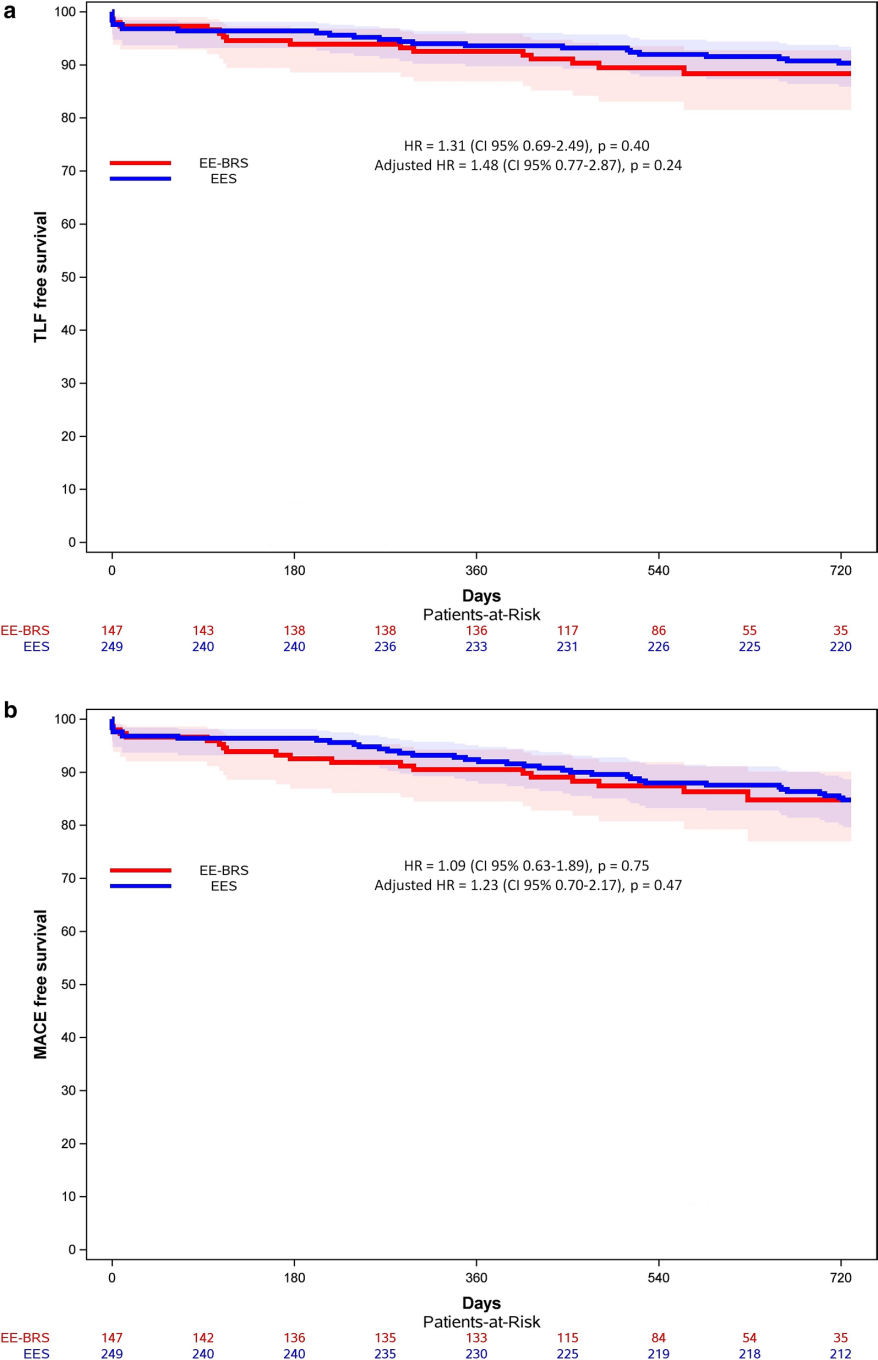
Shown are the Kaplan–Meier curves with 95% confidence intervals in function of **a** target lesion failure free survival and **b** major adverse cardiac events free survival at 2-year follow-up. A log-rank test did not prove significant differences between both treatment groups. In a Cox regression model with adjustment for age, insulin-treated diabetes mellitus, number of treated vessels and total treated length, treatment with everolimus-eluting bioresorbable scaffolds could not be identified as significant risk factor for both target lesion failure and major adverse cardiac events. *TLF* target lesion failure, *EE-BRS* everolimus-eluting bioresorbable scaffolds, *EES* everolimus-eluting stents, *HR* hazard ratio, *CI* confidence interval, *MACE* major adverse cardiac events

**Fig. 2:**
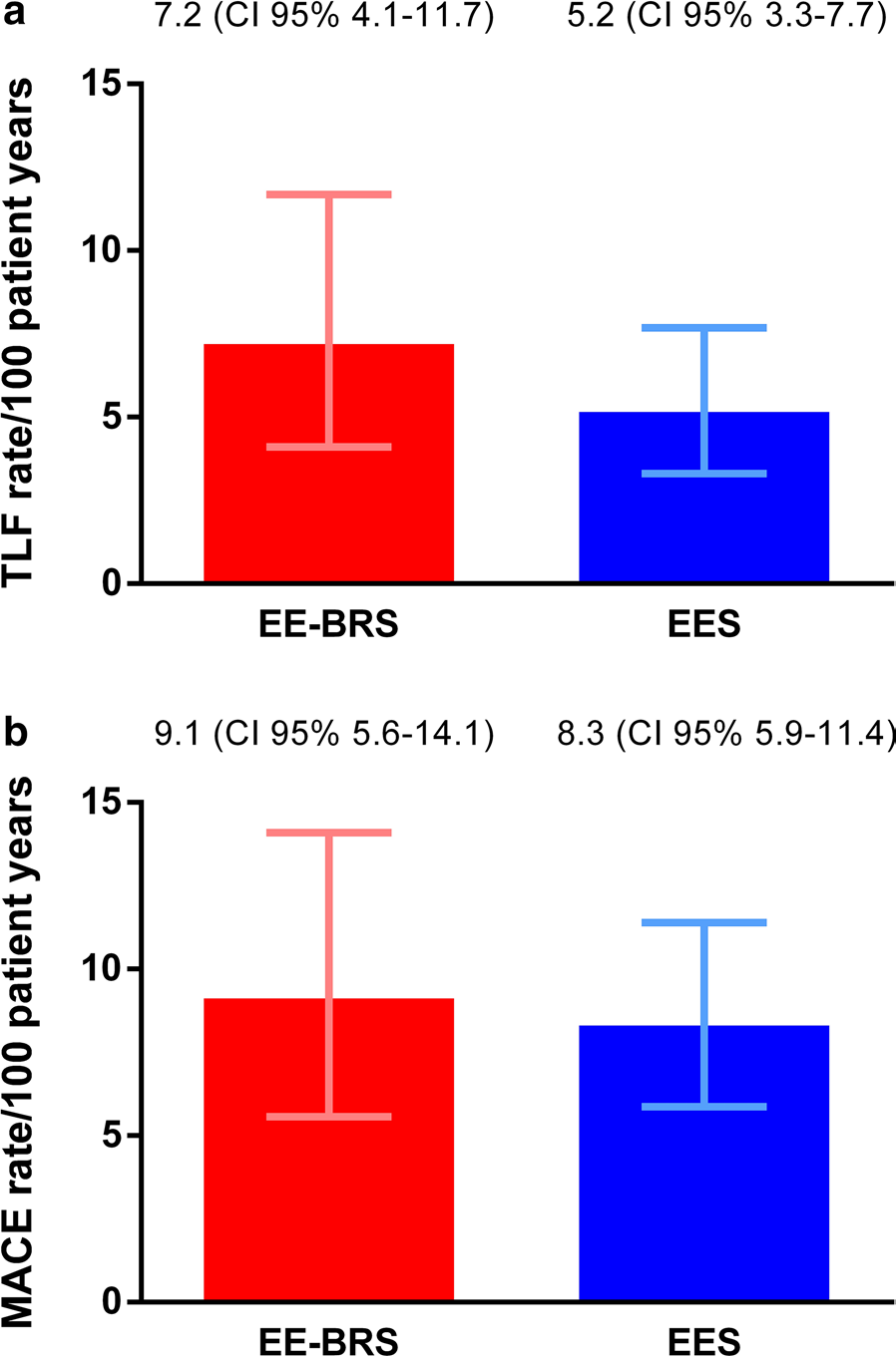
Shown are the event rates per 100 patient years for the composite endpoints **a** target lesion failure and **b** major adverse cardiac events for both treatment groups (red and blue bars). In addition, the 95% confidence intervals are also presented (red and blue brackets). No significant differences were found between both treatment groups. *TLF* target lesion failure, *CI* confidence interval, *EE-BRS* everolimus-eluting bioresorbable scaffolds, *EES* everolimus-eluting stents, *MACE* major adverse cardiac events

Follow-up was available for all but 3 patients who were lost to follow-up. Total available follow-up was 222.6 PY for the EE-BRS and 464.9 PY for the EES arm. Clinical outcomes are reported in Table 3 and Figs. 1 and 2. In all clinical endpoints, there was no significant difference between EE-BRS and EES. TLF event rates were 7.2 vs. 5.2 events per 100 PY (p = 0.39) (adjusted HR 1.48, 95% CI 0.77–2.87; p = 0.24). The rates of MACE were 9.1 vs. 8.3 events per 100 PY (p = 0.83) (adjusted HR 1.23, 95% CI: 0.70–2.17; p = 0.47). No significant difference was observed in any individual component of these composite endpoints. ST rates were 0.9 vs. 0.6 per 100 PY (p > 0.99). Table 4 present the results of the multivariate Cox regression model for TLF and MACE. After correction for age, insulin-treated diabetes mellitus, multiple vessel treatment and total treated length, we found again no significant difference between EE-BRS and EES. Nevertheless, multivariable analysis revealed borderline non-significant relations between insulin-treatment and TLF (p = 0.06) and between patient age and MACE (p = 0.06).

**Table 3:** Safety and efficacy outcomes at follow-up

Endpoints and clinical events	EE-BRS (n = 147)		EES (n = 249)		P-value log rank	P-value PY event rate
	% (n) KM	100 PY (95% CI)	% (n) KM	100 PY (95% CI)		
TLF^a^	11.7 [16]	7.2 (4.1–11.7)	9.7 [24]	5.2 (3.3–7.7)	0.40	0.39
Cardiac death	3.4 [5]	2.1 (0.7–5.0)	4.4 [11]	2.3 (1.1–4.1)	0.84	>0.99
TV-MI	3.6 [5]	2.2 (0.7–5.1)	2.8 [7]	1.5 (0.6–3.1)	0.69	0.69
TLR	5.5 [7]	3.1 (1.2–6.3)	3.3 [8]	1.7 (0.7–3.3)	0.23	0.36
MACE^b^	15.2 [20]	9.1 (5.6–14.1)	15.3 [38]	8.3 (5.9–11.4)	0.75	0.83
All-cause death	3.4 [5]	2.1 (0.7–5.0)	6.8 [17]	3.5 (2.1–5.6)	0.35	0.45
Any MI	4.9 [7]	3.1 (1.3–6.4)	3.2 [8]	1.7 (0.7–3.3)	0.40	0.36
TVR	9.3 [11]	4.9 (2.5–8.8)	6.6 [16]	3.4 (2.0–5.5)	0.29	0.46
ST^c^	1.4 [2]	0.9 (0.1–3.1)	1.2 [3]	0.6 (0.1–1.8)	0.90	>0.99
Early	[2]		[2]			
Acute	(0)		[2]			
Subacute	[2]		(0)			
Late	(0)		[1]			
Very late	(0)		(0)			
Definite	[1]		[1]			
Probable	[1]		[2]			

Shown are the clinical outcomes represented as endpoints and clinical events. Three patients were lost to follow-up in the everolimus-eluting bioresorbable scaffolds group. The results are presented by 2-year Kaplan–Meier estimates and are also reported in event rates per 100 patient years with 95% confidence intervals to adjust for the variable time to follow-up between both groups. A p-value < 0.05 was considered as statistically significant; no significant differences were found between both treatment groups at follow-up

^a^Target lesion failure was defined as a composite of cardiac death, target vessel myocardial infarction and clinically driven target lesion revascularization

^b^Major adverse cardiac events were defined as a composite of all-cause death, any myocardial infarction and clinically driven target vessel revascularization

^c^Device thrombosis was defined as early if observed between 0 and 30 days after index procedure, including a further distinction between acute ≤ 24 h and subacute > 1–30 days. Device thrombosis was defined as late if ≤ 1 year and as very late if > 1 year

*EE-BRS* everolimus-eluting bioresorbable scaffolds, *EES* everolimus-eluting stents, *PY* patient years, *KM*  Kaplan–Meier, *CI* confidence interval, *TLF* target lesion failure, *TV-MI* target vessel myocardial infarction, *TLR* target lesion revascularization, *MACE* major adverse cardiac events, *MI* myocardial infarction, *TVR* target vessel revascularization, *ST* device thrombosis

**Table 4:** Multivariate Cox regression models for target lesion failure and major adverse cardiac events

A. Variable for outcome TLF	Hazard ratio	95% CI	P-value
Age at device implantation	1.03	0.99–1.06	0.12
Insulin-treated diabetes mellitus	1.82	0.97–3.41	0.06
Number of treated vessels	1.17	0.47–2.91	0.74
Total treated length	1.00	0.99–1.02	0.84
EE-BRS vs. EES	1.48	0.77–2.87	0.24

B. Variable for outcome MACE	Hazard ratio	95% CI	p-value
Age at device implantation	1.03	1.00–1.05	0.06
Insulin-treated diabetes mellitus	1.40	0.82–2.37	0.22
Number of treated vessels	1.57	0.77–3.22	0.22
Total treated length	1.00	0.99–1.01	0.88
EE-BRS vs. EES	1.23	0.70–2.17	0.47

Section A. Multivariate Cox regression model for target lesion failure adjusted for age, insulin-treated diabetes mellitus, number of treated vessels, total treated length and destined treatment group

Section B. The same model calculated for major adverse cardiac events. Risk factors are given in hazard ratios with 95% confidence intervals with corresponding p-values

A p-value < 0.05 was considered as statistically significant. No significant differences between both everolimus-eluting bioresorbable scaffolds and everolimus-eluting stents treatment groups were ascertained. Insulin-treatment for diabetes mellitus was the only variable that showed a trend as predictor for target lesion failure as was age for major adverse cardiac events

*TLF* target lesion failure, *CI* confidence interval, *EE-BRS* everolimus-eluting bioresorbable scaffolds, *EES* everolimus-eluting stents, *MACE* major adverse cardiac events

## Discussion

This analysis of pooled patient-level data, comparing EE-BRS with EES for treatment of CAD in patients with diabetes mellitus, showed similar event rates in both treatment groups for the device-oriented and the patient-oriented composite endpoint. Importantly, safety outcomes were also similar, including cardiac death and TV-MI. A multivariable analysis for the composite clinical endpoints TLF and MACE confirmed the absence of a significant difference between both groups.

### Comparing EE-BRS to EES in a wider perspective

Despite advances in interventional therapies and the implementation of new-generation DES, diabetic patients still have worse angiographic and clinical outcomes compared to nondiabetic patients undergoing PCI [21]. Nevertheless, as shown in the EXCEL trial, the relative 30-day and 3-year outcomes of PCI with EES compared to CABG were consistent in diabetic and nondiabetic patients with left main disease with low or intermediate SYNTAX score [22]. Other factors like renal failure and hemodialysis as well as in-stent restenosis, both occurring more frequently in diabetic patients, might influence safety outcomes in this group [23].

In the present analysis, diabetic patients treated with EES showed clinical event rates similar to those of the EES-treated diabetic patients in a pooled analysis of the SPIRIT and COMPARE trials [6]. Furthermore, in our patients with diabetes mellitus the efficacy and short- and long-term safety of EE-BRS treatment were favorable and comparable to previous studies [24–27]. The performance of EE-BRS and EES have been compared in non-exclusive diabetic populations. Despite some promising early results, long-term assessment revealed no advantage for treating all-comers with EE-BRS, but revealed a higher incidence of TV-MI and ST, and a greater angiographic late lumen loss [28–32]. The findings of these trials led to the current withdrawal and utilization of EE-BRS and suggest a need for refined bioresorbable devices and a modified duration of DAPT (to correspond with scaffold resorption). Yet, it should be considered that in these previous trials operator experience with EE-BRS implantation was limited and that there was no requirement to follow a formal EE-BRS implantation protocol such as the pre-dilatation, sizing, and post-dilatation (PSP) approach, which in other studies improved safety outcomes [33–35]. Interestingly, a 3-year landmark analysis of the ABSORB trials showed a significant improvement in composite safety outcomes beyond the resorption of the EE-BRS [36, 37].

Nevertheless, previous trials did not focus exclusively on diabetic patients. Patients with diabetes mellitus represent a high-risk population that theoretically might show particular benefit from being treated with bioresorbable devices. This is because such devices ‘disappear’ over time, which allows for repeated PCI procedures in the same coronary segment without resulting in multiple metallic mesh layers. Furthermore, the EE-BRS does not cause an incessant stimulation of the diabetes-related vascular inflammation, which may be the case in the permanent presence of certain durable polymers. Therefore, it is conceivable that after PCI with EE-BRS long-term results might be more favorable in patients with diabetes mellitus (despite short and medium-term results similar to EES).

### Revascularization in insulin-treated diabetic patients

In our current all-diabetic patient population, treatment with insulin showed an, albeit statistically non-significant, trend towards prediction of TLF. Such relation was observed in the diabetes mellitus subgroup analysis of the ABSORB trials and in a pooled analysis of the SPIRIT and COMPARE trials; both analyses found insulin-treatment to be a risk factor for TLF after PCI with either EE-BRS or EES [6, 24]. The increased event risk in insulin-treated patients may be attributable to the generally longer history of diabetes mellitus, during which the diabetes-induced chronic vascular inflammation stimulates the progression of atherosclerosis, alters plaque composition and promotes the development of advanced lesions. It is plausible that the severity and duration of diabetes mellitus is related to the risk of cardiovascular complications and it appears reasonable to consider this when choosing a coronary revascularization strategy.

### Patient selection for EE-BRS treatment

Younger patients with non-insulin-treated diabetes mellitus and CAD of limited extent may be the most suitable candidates for treatment with EE-BRS (as an alternative to EES), as they might have the greatest benefit from the bioresorbable nature of the device. Furthermore, younger patients have a lower bleeding risk than elderly patients, which is beneficial considering that a prolonged DAPT regimen may be indicated following PCI with EE-BRS. Nevertheless, further dedicated research is required to assess the usefulness of EE-BRS in younger patients. Most likely, such studies will test novel thinner-strut bioresorbable scaffolds that recently became available for research purposes and these studies should include a long-term follow-up [38].

## Limitations

The hypothesis-generating findings of the present post hoc analysis should be interpreted carefully in the light of several limitations, including the intrinsic limitations of any comparison between multiple patient cohorts from different studies. Low cholesterol, and in particular values of remnant-like particle cholesterol below 0.5 mmol per liter have shown to be a predictor of freedom from in-stent restenosis in diabetic patients for all types of stents [39]. In our study detailed patient specific cholesterol values were unobtainable, however a dichotomic hypercholesterolemia (treated) was available and similarly distributed between both groups. Profound data concerning the status of diabetes mellitus and medication prescription were unavailable for a substantial share of the included patients. Yet, all study participants were treated in the same geographical region and they received the same concomitant medication reflecting the international guidelines. Although most baseline clinical characteristics were fairly comparable, there were some differences in angiographic lesion characteristics. Hence, a multivariable analysis was performed, striving for adjustment of known confounders. Due to the population size, this study was not powered to detect significant differences in the composites of the endpoints, particularly low-incidence events like mortality and ST, therefore these results should be interpreted with necessary caution. As we decided to share our results now as debate concerning the future of bioresorbable devices continues, this study does not provide sufficient information about differences between these devices in the phase beyond scaffold resorption in the majority of the EE-BRS patients. To correct for the variable time to follow-up, results were presented in PY which theoretically may obscure time-to-event related adverse outcomes. To minimize this limitation we also reported time-to-event analysis and the nominal incidence of ST. While the operators had considerable experience with implanting EE-BRS, the use of the PSP approach and intracoronary imaging were not mandatory according to the study protocol, while this might have improved clinical outcomes. Finally, as the current EE-BRS generation has been withdrawn from daily practice due to the comprised safety outcomes, the clinical implication of our results should be interpreted in the light of newer bioresorbable devices which have been introduced recently.

## Conclusion

In this patient-level pooled analysis of diabetic patients from 3 prospective clinical trials, EE-BRS showed similar clinical outcomes to EES. These results encourage further prospective long-term follow-up research with novel bioresorbable scaffolds in this patient population.

## Data Availability

The dataset used and/or analyzed in the current manuscript was pooled from 3 individual clinical trials. Consequently, the authors of this manuscript do not own the underlying patient-level data. Upon reasonable request, data may be available from the respective organizing centers, but not without formal permission of the principal investigators and the steering committees of each of the separate trials. Corresponding author for any data request is E. Kedhi, the senior corresponding author of this manuscript.

## Abbreviations

CABG: Coronary artery bypass grafting
CAD: Coronary artery disease
CI: Confidence interval
DAPT: Dual antiplatelet therapy
EE-BRS: Everolimus-eluting bioresorbable scaffolds
EES: Everolimus-eluting stents
HR: Hazard ratio
MACE: Major adverse cardiac events
MI: Myocardial infarction
PCI: Percutaneous coronary intervention
PSP: Pre-dilatation, sizing, and post-dilatation
PY: Patient years
ST: Device thrombosis
TLF: Target lesion failure
TLR: Target lesion revascularization
TV-MI: Target vessel myocardial infarction
TVR: Target vessel revascularization
